# Reflection on whether Chat GPT should be banned by academia from the perspective of education and teaching

**DOI:** 10.3389/fpsyg.2023.1181712

**Published:** 2023-06-01

**Authors:** Hao Yu

**Affiliations:** Faculty of Education, Shaanxi Normal University, Xi'an, Shaanxi, China

**Keywords:** Chat GPT, academic ban, educational digital transformation, integration of educational technology, theoretical debate

## 1. Introduction

The new generation of artificial intelligence represented by Chat GPT (Generative Pre-trained Transformer) is driving the innovative development of intelligent technology into a new historical stage. This technology is not only profoundly influencing and shaping the production, life, and communication modes of the entire society, but also fundamentally reshaping society and humanity itself (Hill-Yardin et al., 2023). Since the emergence of Eliza, chatbots based on artificial intelligence generated content (AIGC) technology have been continuously developing and innovating. The emergence of chatbots such as Microsoft Xiaoice and Google Siri, as well as the continuous upgrading of technologies such as Chat GPT, marks the entry of this development process into a spiral upward historical stage (Rahaman et al., 2023). In the future, more advanced technologies may appear, which may change the form of chatbots or the way people chat, in order to improve user experience. The biggest feature of Chat GPT and other similar products is that they have established content generation rules, which can be collectively referred to as AIGC products. These products are closely integrated with people's daily lives, able to establish a deep connection with each individual, thus profoundly influencing people's behavior patterns and prompting continuous innovation in learning patterns.

When Chat GPT was launched on November 30, 2022, it was considered an unprecedented technological revolution. The chatbot model was developed by OpenAI and attracted over one million users in just five days, generating widespread attention and discussion globally. The release of Chat GPT sparked a global frenzy of development, with companies from the technology and internet sectors, as well as physical and traditional enterprises, all joining the ranks of diversified application product development based on Chat GPT. Recently, Microsoft launched a new Chat GPT search engine and Edge browser, and plans to incorporate it into all of its business sectors, including Bing, Office, GitHub, and Azure. Google also announced a chatbot service, Bard, and collaborated with the AI startup Anthropic to develop an AI assistant, Claude, to enhance user experience (Strowel, 2023). In March 2023, Chinese internet giant Baidu completed the internal testing of its product, the ERNIE Bot, and opened it to some users (Stokel-Walker, 2022). This series of application and product development marks the rapid popularization and application of Chat GPT technology, as well as the continued deepening of artificial intelligence technology in various fields.

ChatGPT (GenerativePre-trainTransformer) is a large language model (LLM) based on the GPT-3.5 architecture. It uses the logic of “big data + big computing power + algorithm = intelligent model,” which can extract valuable information from massive text data, and through training, generate more complex and humanized works to output answers and feedback in the form of text, So as to realize multi-round human-computer dialogue with the help of natural language (Cheng et al., 2023). The development of Chat GPT technology holds the promise of completely upending human lifestyles, bringing about unprecedented digital experiences and enriching people's lives. However, this technology also presents potential negative impacts. With the maturation and application of Chat GPT technology, it may replace many jobs that require tasks beyond the capability of artificial intelligence, leading to an increase in unemployment rates and negatively affecting the overall economy. Additionally, the application of Chat GPT technology may also increase people's reliance on artificial intelligence, thus diminishing human thinking and judgement abilities.

Aside from its effects on the job market, Chat GPT technology has also drawn significant attention from academic circles. Compared to conventional search engines and intelligent chatbots that offer mechanical responses solely based on keyword searches, Chat GPT breaks free from the limitations of existing indexing, retrieval, and sorting models by accurately understanding the semantic meaning and intent of questions, providing organized and coherent human-like feedback, and modifying answers based on user feedback (see Table 1) (Deng and Lin, 2023). Chat GPT passed 92.5% of the theory-of-mind test tasks, achieving a mental capacity equivalent to that of a 9-year-old child. The large language model represented by Chat GPT exhibits the ability to think and answer questions like a human being, manifesting a creative capacity that was previously unavailable to artificial intelligence, marking a qualitative leap from quantitative change (Kosinski, 2023).

**Table 1:** Comparison of educational applications of different artificial intelligence technologies.

	**Traditional educational chat robots**	**ChatGPT**
Search mode	Keyword based retrieval	Based on large-scale corpus learning
Response quality	Answering questions mechanically	Similar manual feedback
Answer scope	Limited answerable questions	Significantly expanding the scope of answerable questions
Understanding level	Context understanding not supported	Ability to understand context
Iterative ability	Cannot iterate based on user feedback	Ability to iteratively optimize based on user feedback

However, there is cause for concern as one study has shown that peer reviewers can only identify 63% of abstracts written using Chat GPT, indicating that such instances of academic fraud may precipitate a reputation crisis within academia (Thorp, 2023). Therefore, the development of Chat GPT technology should be subject to careful regulation to ensure that its applications do not have adverse societal impacts. Furthermore, the application of Chat GPT technology must consider how to integrate with human thinking and judgment to achieve optimal results. Given the nature of the topic and the intent of the article, this paper is an opinion-based essay that utilizes macro-level thinking and integrated logical analysis as a methodological foundation to discuss relevant viewpoints and issues.

## 2. Development: from exploring artificial intelligence to Chat GPT plus

In 1956, a group of scientists gathered at Dartmouth College to explore the possibility of automation through machine learning and other technologies that transcended the cognitive domain of humans. Although the conference did not reach a consensus, it sparked a profound transformation in the field of artificial intelligence (AI), which had a significant impact on subsequent generations (Howard, 2019). Over time, AI has undergone three major waves of development. The emergence of the Turing test in the 1950s and 1960s marked the arrival of the first wave of AI enthusiasm. However, computer technology, programming, and algorithm theory at the time were still inadequate to meet the needs of AI development (Sejnowski, 2022). In 1982, with the advent of the Hopfield neural network and the BT training algorithm, AI entered its second peak. The development of technologies such as speech recognition and translation, as well as Japan's Fifth Generation Computer Project, further promoted the development of AI. Nevertheless, due to limitations in hardware and algorithms, AI development once fell into a trough (Garvey, 2019). Since the beginning of the 21st century, the rapid development of information technology such as big data and cloud computing has facilitated the emergence of four key drivers: massive parallel computing, big data, deep learning algorithms, and AI chips, bringing unprecedented opportunities for the development of AI. Since 2006, AI has entered the third wave of deep learning, which is continuing to grow and develop (Kersting, 2020). At the end of 2022, Chat GPT developed by OpenAI was hailed as the most advanced intelligent machine closest to passing the Turing test, ushering in a new, vibrant era of artificial intelligence.

In 2018, OpenAI's developers proposed the GPT-1 model, which uses the Transformer model to automatically learn and train existing text corpora, including grammar, semantics, idioms, and context information without external interference. This innovative natural language processing technology aims to achieve language algorithm models that are more akin to human expression of thought (Finnie-Ansley et al., 2022). Subsequently, in 2019, OpenAI released the GPT-2 model, which is based on the core ideas of the GPT-1 model but employs more Transformer decoders and richer corpora to improve training efficiency and accuracy (Henrickson and Meroño-Peñuela, 2022). In 2020, OpenAI introduced the GPT-3 model with a significantly increased number of parameters, thereby exhibiting superior performance in dialogue generation, text summarization, machine translation, and other tasks. The introduction of these models has injected powerful impetus into the development of natural language processing and brought new opportunities and challenges for future natural language processing technology development (Chan, 2022).

Chat GPT is a large-scale language model based on the GPT-3.5 architecture, officially launched in November 2022. The model employs massive datasets, powerful computational resources, and efficient algorithms to construct an intelligent model that can extract valuable information from vast amounts of textual data and generate more complex and human-like works. Chat GPT can output answers and feedback in text form, enabling natural multi-turn human-computer interaction. Additionally, Chat GPT can convert user input into numerical sequences and use the model to analyze the data, explore its background information, and better understand the user's intent. The introduction of Chat GPT will bring more intelligent and efficient solutions to the field of human-computer interaction, providing new opportunities and challenges for the future development of natural language processing technology (Abdullah et al., 2022).

With the continuous update and iteration of technology, OpenAI released the latest generation of large-scale multimodal language model, GPT-4, on March 14th, 2023. Compared with the GPT-3.5 model, GPT-4 not only can receive image and text input but also has greatly enhanced reasoning ability, understanding of complex issues, and code writing capability. At the same time, GPT-4 has achieved breakthroughs in image recognition, text input limitations, answer accuracy, and other aspects. GPT-4 can handle more detailed instructions, generate more diverse and creative texts, and perform more reliably and creatively. For example, in simulating the American Bar Exam, GPT-4 ranked in the top 10%, while GPT-3.5 ranked in the bottom 10% (OpenAI, 2023). Globally, Chat GPT Plus based on GPT-4 has caused a revolutionary change and become an unparalleled presence in the field of natural language processing. Chat GPT Plus has the ability to generate up to 20 different types of programming codes and can easily switch between about 30 languages (Gong et al., 2023). This intelligent ability poses significant challenges to human intelligence and brings unprecedented opportunities. The emergence of Chat GPT Plus will have a profound impact on the work and life of billions of people and present significant technological revolution characteristics.

## 3. Academic integrity risks triggered resistance to Chat GPT

As a new artificial intelligence technology, the widespread application of Chat GPT in education has attracted attention and controversies from all sectors of society. Although Chat GPT has significant advantages in improving learning efficiency and promoting communication, its applications also have some negative impacts and potential risks. On the one hand, while Chat GPT is expected to improve the way and efficiency of interpersonal communication, some people worry that this method may have a negative impact on interpersonal relationships. On the other hand, students using Chat GPT to complete assignments may lead to academic dishonesty and cheating behaviors, which have already sparked opposition and resistance from some universities, publications, and scholars. At the same time, the misuse of Chat GPT in scientific research has also aroused strong opposition and concerns within the academic community.

A recent survey revealed that nearly 89% of American college students use Chat GPT to complete homework tasks, with 53% using the tool for writing papers. Additionally, 48% of students use Chat GPT during exams and 22% use Chat GPT to generate paper outlines (McGee, 2023). However, it is worth noting that some students are not only able to successfully complete assignments using Chat GPT but also achieve high scores. Nevertheless, it is difficult for teachers to determine whether students are using Chat GPT, which has a negative impact on students' over-reliance on this tool, gradually causing them to lose their ability to think critically, explore, verify, and summarize actively. If this trend continues, it will greatly affect students' learning outcomes and development (Kasneci et al., 2023).

As a result, teachers at some North American universities face enormous pressure in course evaluations and have to prohibit students from using Chat GPT tools. Furthermore, to prevent the proliferation of artificial intelligence assignments, the New York City Department of Education announced in January 2023 that students are prohibited from using this tool for plagiarism (Lund and Ting, 2023). In Australia, New South Wales was the first region to restrict students from using Chat GPT, and Queensland, Tasmania, and Western Australia have taken similar measures to ban the use of Chat GPT in public schools to ensure educational quality (Deshpande and Szefer, 2023).

With the widespread use of Chat GPT, many schools worldwide have implemented measures to limit or prohibit its use. For example, Seattle public schools banned the use of Chat GPT in January 2023, and Sciences Po in Paris emphasized the prohibition of scholars using Chat GPT or other artificial intelligence technologies in undisclosed ways (Zhou et al., 2023). RV University in Bangalore, India, issued a strict ban that explicitly prohibits students from using Chat GPT to complete tasks, participate in exams, or laboratory tests (Yadava, 2023). The University of Hong Kong strongly requires students not to use artificial intelligence tools such as Chat GPT without written permission from teachers; otherwise, it will be regarded as plagiarism. The implementation of these measures aims to ensure that students use Chat GPT tools correctly, avoid over-reliance and abuse, and safeguard academic integrity and education quality (Chan and Hu, 2023).

In early April 2023, a highly anticipated joint letter attracted widespread attention from society. The letter was signed by many well-known figures, including Musk, Turing Award winner Yoshua Bengio, Stability AI CEO Emad Mostaque, NYU Professor Marcus, and Yuval Noah Harari, the author of “*Sapiens*.” These signatories expressed in the letter that AI systems that compete with humans' intelligence may pose profound risks to society and humanity (Samuel, 2023). Therefore, they called for a 6-month suspension of the use of AI technology and the cessation of developing large models like GPT-5. This joint letter has sparked broad discussions and controversies in society. Some people believe that the development of AI technology should be supported and encouraged rather than restricted and hindered, while others believe that the rapid development of AI technology may bring enormous challenges and risks to humanity, which needs to be handled with caution.

According to media reports, a recent study was published in the journal “*Scientific Public Library Digital Health*.” The study used Chat GPT, an AI model that has not received any medical training, to participate in the United States Medical Licensing Examination (USMLE), and achieved an accuracy rate close to or reaching the passing level (Doshi et al., 2023). In addition, another study used AI technology to detect the reliability of experimental data, and researchers successfully wrote and published academic papers with the help of AI (Bai et al., 2022). During this process, the authorship of Chat GPT raised concerns from well-known scientific journals. The magazine “*Science*” has reconsidered its publishing strategy and made corresponding adjustments (Thorp, 2023). However, editors of “*Nature*” expressed concern about the potential negative impact of Chat GPT on scientific transparency (Nature, 2023).

As an artificial intelligence technology, the application of Chat GPT in education has attracted the attention of scholars. However, some scholars have expressed concerns about its feasibility and potential negative impacts. Scholars such as Alshater point out that Chat GPT faces various challenges, including dependence on data quality, limitations on knowledge scope, exacerbation of ethical issues, and risks of technical dependence and misuse (Alshater, 2022). Scholars such as Baidoo-Anu believe that the application of Chat GPT in education may lead to problems such as lack of communication, limited understanding ability, inaccurate training data, lack of innovation, insufficient understanding of context, and privacy leakage (Baidoo-Anu and Owusu Ansah, 2023). In addition, scholars such as Qadir also pointed out that Chat GPT and other generative AI systems also have biases and erroneous information, bringing serious moral risks (Qadir, 2022). These scholars unanimously believe that to make Chat GPT play a positive role in education, multiple challenges need to be addressed, including data quality, knowledge reserves, privacy protection, and ethical issues. In addition, it is crucial to strengthen the cooperation between AI and human teachers, fully utilize its advantages, and avoid potential negative impacts. Overall, scholars generally believe that a comprehensive evaluation of the application of Chat GPT in education is needed to ensure its contribution to education and minimize potential negative impacts.

## 4. Supporting education and teaching: some scholars advocate the integration of Chat GPT into the educational ecosystem

In the field of education, Chat GPT is a powerful tool that can be used to create educational content and assist language learning. This tool can automatically generate various texts, including papers, abstracts, and textbooks, with little human intervention, making it an important resource for educators and students. By utilizing artificial intelligence technology, educators can incorporate Chat GPT as part of a diversified teaching tool to achieve a more interesting and innovative teaching experience. Additionally, students can use Chat GPT for self-inquiry, further exploring knowledge points and constructing a genuinely intelligent educational system. Therefore, there are many voices supporting the application of this intelligent tool in education. However, it should be recognized that the application of Chat GPT in the field of education also faces many challenges, such as data quality, knowledge reserves, privacy protection, ethical issues, etc.

At the University of Cambridge, Professor Bhaskar Vira pointed out that university students should fully utilize artificial intelligence technology, such as Chat GPT and other new tools, and should not be limited. These technologies can help students better master knowledge and improve learning efficiency. However, to ensure that students adhere to academic integrity when using these new technologies, schools need to make appropriate adjustments to teaching methods and examination standards (Stephens, 2023). Similarly, Professor John Villasenor at the University of California allows students to use Chat GPT in assignments. But more importantly, it is essential to teach students how to use these technologies correctly and effectively, to ensure that their learning process is meaningful and efficient (Villasenor, 2023). Compared to restricting students from using these AI tools to save time and effort, it is a better choice to integrate these tools into the education system, allowing students to learn and use them in a correct and responsible environment. However, it is essential to balance the rights of students to independently use these tools with the requirements of academic integrity, so that students can use these tools properly and responsibly.

An article published in the journal “*Nature*,” entitled “Chat GPT: Five Priorities for Research,” emphasizes the benefits of embracing artificial intelligence and argues that Chat GPT technology can significantly reduce the workload of researchers, allowing them to devote more time and energy to conducting new experiments, promoting innovation, and achieving breakthroughs in multiple fields. However, the article also points out that Chat GPT faces obstacles related to bias, provenance, and accuracy that need to be overcome (van Dis et al., 2023). Therefore, only by addressing these challenges can Chat GPT truly realize its enormous potential. In practical applications, the advantages of Chat GPT technology are evident. It can automatically generate natural language, helping researchers to quickly complete literature reviews or answer questions, thereby freeing up their time and energy to engage in more creative and high-value research work. However, Chat GPT also faces some challenges, such as bias and provenance issues, that need to be addressed. Overcoming these challenges will lead to the gradual development and growth of Chat GPT technology. Chat GPT will become an important tool for future research, providing scientists with more time and energy to explore new areas and undertake more meaningful research work.

## 5. Is it an opportunity?—A feasibility analysis of artificial intelligence + education

Before discussing whether AI should be academically banned, it is necessary to carefully examine the challenges currently faced by education and the significant impact of artificial intelligence on education. Currently, there are many problems in the field of education from the international perspective, including unequal distribution of educational resources, unstable education quality, insufficient education content, outdated teaching methods, excessive student workload, and imperfect education evaluation system. These problems severely restrict the fairness and quality of education, hindering its progress and development.

**Figure 1:**
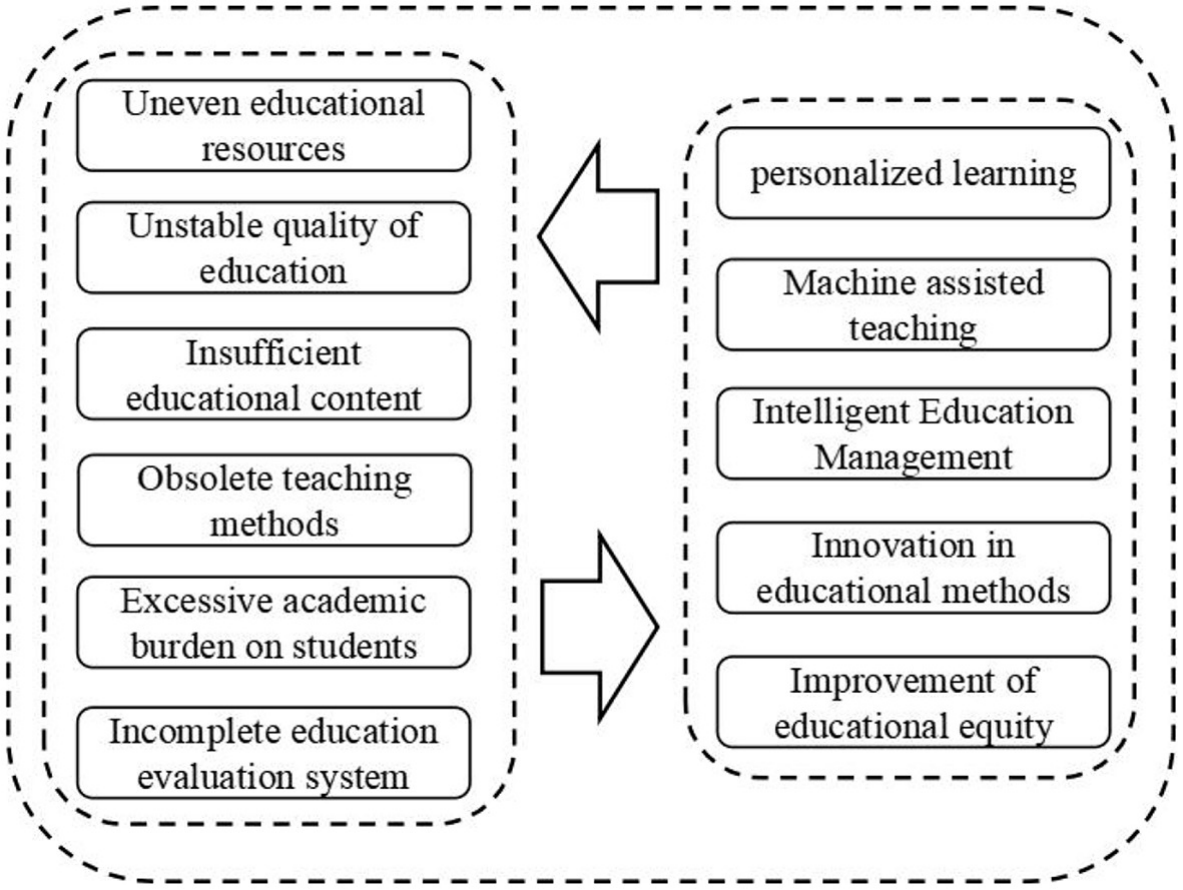
Education issues and the promotion of artificial intelligence in education.

Among them, the unequal distribution of educational resources manifests as a significant gap in educational resources between urban and rural areas or regions. At the same time, there is also an oversupply of educational resources in some areas while others face a shortage of educational resources (Holmqvist, 2019). The instability of education quality shows that some schools have a high-quality education system, but others lack proper educational resources. This has led to the problem of “good/bad schools.” In addition, insufficient education content cannot meet the needs of social development and lacks the ability to cultivate students' comprehensive qualities. The lack of innovative teaching methods fails to fulfill students' demands, and excessive student workload affects their physical and mental health due to the influence of the education examination system. The imperfect education evaluation system with inconsistent evaluation standards fails to reflect students' actual level comprehensively and objectively. Therefore, before discussing whether AI should be academically banned, it is essential to fully consider the relationship between artificial intelligence and education, and combine it with the current state of education to better evaluate the impact of AI on education (see Figure 1).

Artificial intelligence (AI) plays a crucial role in the field of education. On the one hand, AI provides abundant information resources such as online learning and virtual laboratories, enabling students to learn in a broader and more open environment. Additionally, technological advancements have given rise to innovative teaching tools such as multimedia courseware and intelligent teaching systems, which help teachers better manage classroom atmosphere, stimulate students' interests and initiative. On the other hand, technological progress has also brought about more comprehensive and objective assessment methods, including data analysis and intelligent evaluation. These methods can assist teachers in guiding students promptly and improving learning outcomes based on evaluation feedback, thereby enhancing the quality and effectiveness of education.

Overall, AI has brought numerous benefits to the education field, promoting its transformation and fair improvement. However, AI faces some challenges and issues in education, such as data security and privacy protection. Therefore, when promoting the application of AI in education, it is necessary to strengthen technical research and development and management standards to ensure its safe and reliable use in educational practice.

## 6. What should happen regarding education with Chat GPT

Chat GPT has pushed AI thinking to a new level, achieving significant breakthroughs from traditional information exchange methods to intelligent reasoning in a new era. Technology columnist for “*The New York Times*,” Kevin Roose, spoke with dozens of educators who believe that prohibiting the use of Chat GPT in the classroom is misguided. Instead, schools should cautiously adopt Chat GPT as a teaching aid (Roose, 2023). Chat GPT can unleash students' creativity, provide personalized tutoring, and better prepare them for working with AI systems in the future. This technology can better meet students' learning needs, improving their efficiency and grades. Chat GPT can also assist teachers in managing and evaluating students, enabling them to improve course adjustments and teaching quality. However, it is essential to recognize the limitations and risks associated with this technology while establishing corresponding regulations to ensure that students and teachers use Chat GPT correctly, transparently, and develop independent thinking and innovation skills necessary to adapt to future challenges and opportunities (Ienca, 2023).

The author argues that it is not wise to prohibit the use of artificial intelligence technologies such as Chat GPT. As technology advances and artificial intelligence rapidly develops, the AI revolution has arrived. Policies and human controls are no longer sufficient to deal with this change, and effective systems need to be established to regulate the use of AI technology. AlphaGo's success demonstrates that AI has begun to deeply understand and simulate human behavior, achieving significant breakthroughs in fields such as image recognition, automatic driving, and speech recognition. Therefore, these technologies should not be blindly prohibited, but rather regulated effectively to promote the development and application of AI technology. Semantic analysis has always been a difficult problem in the field of artificial intelligence, lacking important technological breakthroughs. However, the latest Chat GPT artificial intelligence large language model technology is gradually replacing traditional high-intelligence behaviors, such as human reading, thinking and writing. This technology brings great convenience to humanity, but also brings some impact, affecting human learning, communication, understanding and behavioral habits. The rapid development of artificial intelligence may affect the career prospects of different groups, so it is necessary to respect and understand the emotions and opinions of different groups and regulate the use of AI technology through effective systems to better serve human society (Ke et al., 2021). Although Chat GPT has improved compared to previous AI products, it still cannot meet the requirements of general artificial intelligence and high-quality academic output. However, AI technology can provide users with knowledge and basic language and text services throughout the entire academic writing process, not only relieving users' time burden, but also improving learning experiences and increasing users' interest and motivation for continuous creation. Therefore, effective systems should be established to regulate and promote the use of artificial intelligence technologies such as Chat GPT to better serve the education and academic research fields.

With the constant development of AI technology, Chat GPT and similar technologies have become the main support for the education and technology sectors. However, the emergence and development of any new thing has two sides. In order to ensure the sustained development of AI technology and its contribution to education, it is necessary to strengthen the construction of relevant laws and regulations and effectively regulate their applications, rather than taking arbitrary actions. Humans need to realize that the application of AI technology brings many benefits to education, but at the same time, there are also potential risks and challenges. For example, over-reliance on AI technology could weaken students' independent thinking and learning abilities, while the use of AI technology may also trigger ethical and privacy concerns (Zhou, 2023). Therefore, while promoting AI technology, rules and regulations on how to apply them must be enforced. Governments and relevant agencies should establish corresponding laws and regulations to protect students' privacy and rights, ensuring that the application of AI technology complies with educational ethics and moral standards. Educators should also strengthen guidance and supervision of students, help them to properly use AI technology, and avoid misuse. In summary, the development of AI technology brings new opportunities and challenges to education. While leveraging its advantages, humans must recognize its potential risks and challenges and take appropriate measures to regulate and manage them, ensuring that AI technology can continue to contribute to education. Additionally, the field needs continuous exploration and innovation to better utilize AI technology to promote the development of education.

On the other hand, the development of technology has always been focused on improving human productivity and quality of life, not destroying the value of technology itself. Humans should cherish the technological achievements created by their predecessors, as they can be directly used to achieve new breakthroughs, revealing the importance of technology. With the constant development of AI technology, humans increasingly rely on it to complete various tasks and need to adopt more advanced technologies to promote future technological innovations, rather than waste time and energy repeating tasks that machines already easily complete. In the face of constantly changing tools and technologies, humans should use them wisely to achieve new breakthroughs, rather than attempting to surpass them. When humans discover that the tasks that machines can easily complete require the help of AI, these tasks will no longer require human participation. Therefore, as the latest achievement of AI, Chat GPT should be fully utilized to maximize its functionality, helping humans to complete tasks, make new breakthroughs, and not be banned or restricted.

The usage of Chat GPT technology has reached billions, indicating a high demand for new technologies in knowledge learning and writing among humans. Therefore, Chat GPT technology has a broad audience base and legitimacy. Although some schools may limit the use of Chat GPT during the K-12 stage, this restriction is understandable since students are at a crucial stage of their cognitive and skill development. However, for higher education students, they should learn to use available resources and tools to complete tasks efficiently and gain better development opportunities. If Chat GPT can perfectly complete assignments assigned by university professors, the latter should reconsider the design of these assignments and evaluate if they could bring substantial improvement to students. As these students enter the job market, they can use Chat GPT technology to complete various work tasks perfectly. This raises a question about whether those jobs need human existence or should be replaced by robots. Therefore, higher education students need different skills and abilities than what Chat GPT possesses and must continue developing themselves to gain a competitive advantage in the job market. Only when humans and technology complement each other can more efficient and convenient solutions be built to meet the needs of learning and social work. Therefore, it is necessary to take full advantage of technologies such as Chat GPT while preserving necessary job positions for humans to adapt to future societal development trends.

With the popularity of distance learning and online education, more and more schools are adopting blended learning methods, combining online and offline courses to improve students' academic performance and efficiency. In this case, artificial intelligence technologies such as Chat GPT become a practical and feasible choice to help students complete tasks better. However, improper use of these technologies may lead to academic misconduct and moral issues. Therefore, schools and educational institutions need to develop policies that regulate students' behavior when using these tools to ensure academic integrity and ethics. Therefore, the author strongly recommends that students should use Chat GPT technology correctly and reasonably, considering it an effective means of improving learning efficiency and quality, and explore and develop continuously for fields where Chat GPT is incompetent. At the same time, governments and schools should strengthen research and development of technologies such as Chat GPT to promote educational reform, break traditional education concepts, teaching models and practices, and provide a better development environment for education in the Chat GPT era.

### 6.1. Digital transformation in education: urgent necessity of new technologies

In the era of thriving AI technology, it is necessary to rethink the focus of education in order to meet the demands of future society. Therefore, educators need to explore which knowledge and abilities are worth cultivating and improving. First, it is necessary to analyze deeply why there is a disconnect between school curricula and social needs, personal development, and future trends. At the same time, problems existing in the school curriculum, such as outdated or impractical content, also need to be examined. Such analysis could help better understand the shortcomings of the current education system, thus providing useful references for educational reform. Based on this, the following three perspectives will be discussed in this article:

#### 6.1.1. Broadening knowledge—New challenges for school curricula

According to data presented by Gilbert Valverde and William Schmidt in the Third International Mathematics and Science Study (TIMSS) report, US fourth-grade students perform well globally; but when they reach eighth grade, their performance falls below the global average. Research shows that compared with countries where academic performance is better, US mathematics and science textbooks are heavier, covering a wider range of topics. This indicates that the themes covered in textbooks are too broad, resulting in lack of depth (Schmidt et al., 1997). Thus, textbooks can only cover surface-level content and cannot delve deeply into more profound knowledge points. One reason for this phenomenon is that textbooks contain too much information. It might be due to stakeholders' interests who hope to include their favorite content in teaching plans, but few people consider which content is truly important and should be included in the curriculum. With the emergence of Chat GPT, the problem of textbook content becomes even more prominent, requiring rethinking and development. Therefore, it is necessary to re-examine the design of textbook content to ensure that the covered knowledge points are in-depth and valuable. Meanwhile, attention needs to be paid to collaborative efforts between all stakeholders in ensuring that the focus of textbook content is on practical application and future needs. Lastly, emphasis should be placed on actual effectiveness evaluation of textbooks to promptly detect and solve existing problems, thus improving education quality.

#### 6.1.2. Outdated content—The awkward situation of education and teaching

In today's society, the rapid development of artificial intelligence technology is profoundly impacting learners' cognitive understanding of learning materials. Particularly in the field of mathematics education, although AI can already perform a variety of mathematical operations from primary school to graduate level, current school curriculums still follow structures from the 19th century or even earlier periods. However, the focus of mathematics classrooms still lies in teaching students how to practice mathematics operations, rather than providing them with perspectives and concepts for deep understanding of mathematics. In order to adapt to the continuous development of AI technology, it is necessary to re-examine the direction and goals of mathematics education.

The internet is a powerful tool for storage and transmission, allowing people to access information anytime and anywhere through smartphones, while artificial intelligence like Chat GPT is a powerful and convenient tool that helps humans better use the internet. However, the knowledge provided by these tools is only superficial and mechanized, which cannot help students deeply understand the surrounding world and operate effectively in it. Obviously, most of this knowledge will be forgotten in daily work and life, so why spend a lot of time learning such knowledge? If educators cannot effectively change the school environment to enable students to explore and practice according to their own interests and needs, they are likely to forget much of the knowledge and skills learned in school. Therefore, educators should re-examine the goals and teaching methods of education based on the development trend of AI technology, to cultivate students' creative thinking and problem-solving abilities, enabling them to better respond to future social and career demands.

#### 6.1.3. Assessment monism—Difficulty achieving objectivity requirement

In the current education system, many schools aim to ensure that their students master the knowledge and skills considered important by experts. However, some schools lack support and encouragement for students to explore their own interests. Instead, they divide teaching content into multiple independent units, teaching them one by one, to ensure comprehensive coverage of all content. Ultimately, students will receive exams to test their mastery of what they have learned. However, Piety (2013) and Abeles and Rubenstein (2015) have pointed out that the design concept of such exams is to objectively evaluate students' understanding of knowledge but it has also led to widespread cheating and the phenomenon of test-oriented education. Educators need to break down complex tasks into a series of simple facts and skills in order to develop these exams. However, this approach sometimes leads to anomalous situations. For example, in an English writing exam, since writing requires creating a large number of paragraphs, objective scoring cannot be achieved. Therefore, teachers usually rely on aspects such as vocabulary, grammar, and editing to grade students' papers. However, this scoring method does not always meet the requirement of objectivity.

Over time, education researchers have developed standardized rating scales and have provided rating training for teachers to enable them to evaluate students' performance more objectively and reliably. Yet with the standards set by standardized rating scales, English teachers found that this type of exam scoring standards and methods were a limitation because they did not consider innovation and complexity. Indeed, many complex abilities cannot be measured by conventional objective assessment techniques, but they play an extremely important role when students face various unknown and vague challenges. Because schools rely too much on simple exam results, some core goals of education may be neglected or even underestimated. Therefore, it is necessary to re-examine the goals of education to ensure that students not only master the knowledge and skills considered important by experts but also develop their interests and talents, and possess innovative and problem-solving abilities.

### 6.2. What kind of talents are worth cultivating?—Turning point of talent development standards

The goal of education is to cultivate students' comprehensive intellectual abilities, including critical thinking, fluent writing, strong logical thinking, accurate language expression, and agile thinking. The cultivation of these abilities is a holistic process and should not be limited to any particular subject. In 1991, the Employment Skills Commission Report (Labour, 1991) released by the US Department of Labor proposed that learners should possess five core competencies: discovering, organizing, planning and allocating resources, cooperating with others, acquiring and applying information, understanding complex systems, and mastering various technologies (Collins, 2017). To adapt to an increasingly competitive environment, Tony Wagner proposed seven crucial survival skills, including critical thinking and problem-solving, influence leadership through network collaboration, agility and adaptability, initiative and entrepreneurial spirit, effective oral and written communication, acquiring and analyzing information, and curiosity and imagination (Vyas, 2018). David Cohen pointed out that the main goal of education is to cultivate students with multiple intellectual abilities, including critical thinking, fluent writing, strong logical thinking, accurate language expression, and agile thinking, which are not limited to any single subject but rather involve overall development (Bona et al., 1985). In addition, Bernie Trilling and Charles Fadel think that necessary skills can be divided into three categories: learning and innovation skills such as critical thinking and problem-solving, digital literacy skills such as information and media literacy, and career and life skills such as adaptability and self-direction (Trilling and Fadel, 2012).

**Figure 2:**
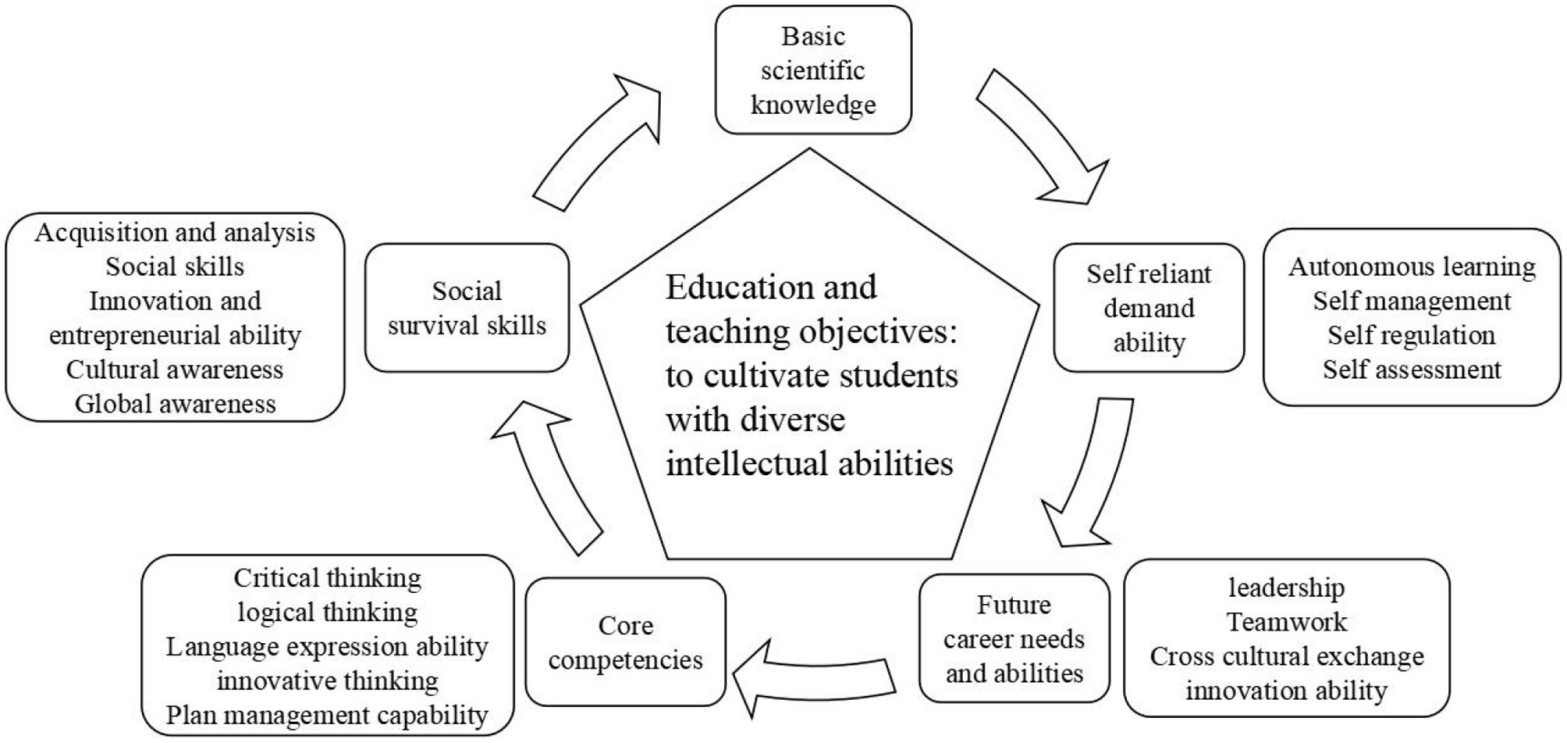
Qualities that students with multiple intelligence abilities should possess.

Combining the above studies, the author believes that with the popularization of artificial intelligence, the goal of education should gradually shift to cultivating students with diverse intellectual abilities, including core competencies, self-reliance needs, future career needs, basic scientific and social survival skills, etc. (see Figure 2). The development of modern society requires students to possess comprehensive and integrated abilities rather than just mastery of a single subject.

### 6.3. Integration of Chat GPT and education: rational thinking for digital transformation

Based on comprehensive analysis of relevant research, the author believes that with the rapid development and widespread application of artificial intelligence technologies such as Chat GPT, the field of education will be significantly impacted (Okaibedi, 2023). The emergence of these technologies will profoundly change traditional educational models and teaching methods, bringing unprecedented opportunities and challenges to education and teaching. Therefore, it is necessary to be prepared in advance, carefully examine the impact of Chat GPT on the field of education, and delve into the risks inherent in artificial intelligence technology (Susnjak, 2022). At the same time, active exploration is needed on how to reconstruct the education ecosystem to cope with the challenges posed by new technologies, and achieve important goals of educational transformation and leapfrog development.

With the continuous evolution and widespread application of artificial intelligence technologies such as Chat GPT, these technologies will have a profound impact on traditional education methods in the field of education. Therefore, it is necessary to think seriously about the impact of Chat GPT on education, and deeply analyze the potential risks of artificial intelligence technology, such as data privacy breaches and algorithmic unfairness (Haque et al., 2022). To ensure the legitimate, fair, and transparent use of these new technologies in education, regulatory and normative measures need to be strengthened. Finally, exploration of new educational models and teaching methods is essential to meet the needs of the new era. The transformation and upgrading of education must be continuously promoted to meet the ever-changing social demands. With the promotion of new technologies, efforts should be made to seek new educational models and teaching methods, create more flexible and efficient educational environments, focus on students' comprehensive development, and improve the quality and effectiveness of education and teaching. To achieve this, educators and learners need to work together to promote the transformation and leapfrog development of education to meet the needs of modern society.

#### 6.3.1. Educational *g*oals

With the rapid development of artificial intelligence technology, the degree of business automation in various industries continues to increase, which poses unprecedented challenges to traditional education and teaching. Existing knowledge and skills are constantly being replaced by new technologies and knowledge. Therefore, the education industry must continuously innovate to adapt to the rapid development of technology.

To this end, the education industry has introduced many new technologies and knowledge to meet the constantly changing learning needs. In the era of Chat GPT-like artificial intelligence, the goal of education has shifted from imparting knowledge, cultivating skills, and providing employment opportunities to adaptive learning (Gao et al., 2022). The education industry should focus on cultivating students' critical thinking, problem analysis, and effective solution-seeking abilities. In addition, students need to learn how to use technology to improve society, master how to use data and analytical techniques for efficient decision-making, and how to effectively identify and judge artificial intelligence. To achieve this goal, schools should focus on developing students' learning ability, enable them to actively explore knowledge and engage in digital learning in the most effective way. Students should be trained to develop good self-control skills, especially in dealing with time, energy, and emotions. Education and teaching should focus on cultivating a new generation of builders with international perspectives and digital skills. Additionally, schools should cultivate students' artistic accomplishments and design skills, enabling them to appreciate beautiful things. Most importantly, students should possess the ability to discover and solve problems, particularly in solving complex problems. Only then can they cope with various challenges in their future careers and become leaders and promoters of the AI era.

In summary, the education industry must continuously innovate, adapt to changes and developments of the times to cultivate new talents with critical thinking, problem analysis, and effective solution-seeking abilities, as well as digital skills and international perspectives. This is an important way to promote the progress and development of AI and human society.

#### 6.3.2. School education

In the field of education, school education plays an irreplaceable role. In terms of curriculum, teachers have the responsibility to cultivate students' critical thinking and effective skills, including mastering artificial intelligence technologies such as Chat GPT (Zhai, 2022). Teachers should also strive to create a safe learning environment, where students can feel confident to explore the potential value of artificial intelligence without fear of failure or blame. Furthermore, teachers should guide students to realize the importance of collaboration and communication, which are essential abilities for achieving mutual success with artificial intelligence. By encouraging students to use artificial intelligence technology to explore and analyze complex situations in the real world, teachers can stimulate their problem-solving skills. To promote the development of artificial intelligence technology, teachers should help students master the basics of programming languages so that they can efficiently construct algorithms.

Driven and catalyzed by artificial intelligence technologies such as Chat GPT, teachers can employ various teaching methods and strategies to provide students with more efficient, interesting, and practical learning experiences (Else, 2023). These teaching methods and strategies include project-based learning, experiential learning, inquiry-based learning, cooperative learning, educational games, and problem-based learning. In project-based learning, teachers can guide students to use artificial intelligence technology to design and complete practical tasks, thereby improving their abilities and skills. In experiential learning, teachers can enable students to deepen their understanding of the trends in artificial intelligence development through practical activities such as coding, programming, and robotics. In inquiry-based learning, teachers can encourage students to explore AI-related issues actively, conduct on-site investigations, and enhance their problem-solving abilities. In cooperative learning, teachers can guide students to explore the latest technologies and applications in the field of artificial intelligence in groups or teams to improve learning outcomes. In gamified learning, teachers can combine learning content with game elements to increase students' interest and motivation. Problem-based learning enables students to master theoretical knowledge by solving real-world problems, thus improving their learning outcomes. At the same time, teachers should also focus on cultivating students' critical thinking and innovation abilities to adapt to challenges in the field of artificial intelligence in the future. In addition, teachers should strengthen communication and cooperation with experts and companies in the field of artificial intelligence to maintain the relevance and practicality of teaching content.

#### 6.3.3. Educational assessment

In the past, learning assessments have focused primarily on testing students' knowledge levels while often neglecting their thinking skills, problem-solving abilities, and process-based performance (Graham, 2022). Modern educational philosophy advocates for incorporating a comprehensive development of core competencies into assessments, prioritizing objectivity and fairness in evaluating results to better reflect students' learning and holistic abilities. Therefore, new assessment approaches should emphasize both internal and external collaboration among students, encouraging them to develop wisdom through more thoughtful consideration, rather than wasting time and effort on tasks that can be completed with basic knowledge and low-level thinking, and thereby helping students develop higher-order thinking skills. The optimization of evaluation mechanisms should focus on the following two points:

On the one hand, process-based evaluation should be given priority. The learning process has profound influences on students' thinking styles, habits, and attitudes, which form the foundation for future development. Therefore, when assessing students' learning outcomes, emphasis should be placed on evaluating the process, not just the result. Students can acquire opportunities for independent thinking, exploration, and practice during the learning process, enabling them to realize their own potential and effectively utilize it to enhance their capabilities. In addition, students can cultivate correct values, gain new insights, better understand the world, and make contributions to their own personal development and social responsibilities. Therefore, evaluations should focus more on the learning process itself rather than just results. Although content-generating AI technologies such as Chat GPT can provide students with relatively accurate results, evaluations should prioritize learning itself rather than just results (Shen et al., 2023). Evaluations should also focus on students' internal and external collaborations in the learning process. Through mutual assistance and communication, students are better able to understand and master learning content while developing cooperative and team spirit. Evaluations should also encourage students to exert their initiative and creativity in collaboration to better develop their thinking and problem-solving abilities. In evaluations, attention should also be given to students' process-based performance abilities, such as critical thinking, innovation, communication, and leadership, to fully reflect students' holistic capabilities.

The evaluation mechanism should ensure fairness and objectivity, and prevent subjectivity and discrimination in the evaluation results. Therefore, evaluations should adopt diverse methods, including classroom performance, homework, exams, project practice, and other aspects, to fully reflect students' abilities and potential. At the same time, evaluations should focus on individual differences among students and not evaluate all students using the same standard. Evaluations should use different evaluation methods and criteria based on students' characteristics and abilities to ensure fairness and objectivity in the evaluation process. In evaluations, emphasis should be placed on students' procedural performance capabilities, rather than solely focusing on the results. Students need to make great efforts during the learning process, constantly reflect, think, and explore. Only after a long journey can students cultivate patience and perseverance to face future challenges with a resilient mindset. Therefore, evaluations should focus more on students' learning processes to help them develop their overall abilities and qualities.

On the other hand, attention should be paid to evaluating critical thinking. Evaluating critical thinking in student learning outcomes assessment has become an indispensable trend. In the past, students' learning outcomes were usually evaluated based on their mastery of knowledge, but this assessment model is no longer suitable for today's rapidly developing era. With the emergence of artificial intelligence products and continuous technological updates, even if students master a large amount of knowledge, it is difficult for them to cope with various challenges in complex environments. Therefore, students' critical thinking skills have gradually become one of the important indicators for evaluating their comprehensive abilities. Critical thinking is a unique core factor of human beings. It not only helps learners better understand and apply knowledge but also enables them to acquire information from the external environment and construct their own mental systems. In contrast, although content-generating AI technology (such as Chat GPT) can help students solve academic problems, even so, it is difficult to guarantee the accuracy and reliability of the results. Therefore, in evaluating students' critical thinking skills, it is necessary to reflect on the results generated by Chat GPT and improve answers based on their own thinking abilities (Iqbal et al., 2022). In this case, evaluating students' critical thinking skills becomes particularly important. Students need to think deeply about questions based on the answers generated by Chat GPT and rely on their own thinking abilities to improve their answers. This evaluation method can comprehensively and accurately reflect students' abilities and levels and help them better adapt to emerging challenges in the future.

## 7. Conclusion and prospects

One of the developmental trends in the field of education in the future is the deep integration of education with artificial intelligence (AI) technology. AI technologies, such as Chat GPT, possess intelligent and automated characteristics that can play an important role in the education sector. In the future, AI technology will be widely applied in fields such as personalized learning, virtual education, online classrooms, educational intelligent management, and science and technology education, providing students with efficient, personalized, and comprehensive educational services. The deep integration of education with technology will greatly change the traditional mode of education, improve the efficiency and quality of education, and enable students to better adapt to the development needs of future society. For example, in the area of personalized learning, AI technologies such as Chat GPT can automatically recommend learning content and methods according to the student's learning ability and interests, achieving the goal of personalized learning. In virtual education, AI technologies such as Chat GPT can build virtual educational environments and develop virtual teachers, enabling students to learn anytime and anywhere, improving the convenience and flexibility of learning. In educational intelligent management, AI technologies such as Chat GPT can help educational institutions achieve intelligent management and allocation of teaching resources, improving the utilization and effectiveness of educational resources. These applications will provide students with more comprehensive, efficient, and personalized learning experiences, helping to improve their learning effectiveness and interest. In summary, the deep integration of education with AI technology will greatly change the form of future education, improve the quality and efficiency of education, provide better learning services for students, and help them better adapt to the development needs of future society (Gozalo-Brizuela and Garrido-Merchán, 2023).

However, the widespread application of artificial intelligence technologies such as Chat GPT has brought about numerous ethical challenges and legal risks in addition to its convenience. For example, Chat GPT may be used for academic plagiarism and other forms of intellectual theft, which can have serious negative consequences on academic integrity (Kitamura, 2023). To avoid this situation, researchers and developers of AI need to optimize self-regulatory mechanisms for technologies such as Chat GPT to improve their safety and applicability. At the same time, educators should continue to optimize evaluation mechanisms to ensure fairness and reflect students' knowledge levels and abilities (Gordijn and Have, 2023). Only then can we ensure that Chat GPT has a positive impact on education and achieves long-term sustainability (Kocoń et al., 2023). The deep integration of education with artificial intelligence technologies such as Chat GPT requires joint efforts from AI research and development personnel, educators, and students to achieve optimal educational outcomes. Therefore, it is more important than ever to focus on ethical and legal issues surrounding artificial intelligence technologies and establish sound regulatory mechanisms through joint efforts to promote their safe, reliable, and sustainable application.

## Author contributions

HY wrote the first draft of the manuscript, revised the article, and approved the submitted version.
